# Incorporation of Nanostructured Carbon Composite Materials into Counter Electrodes for Highly Efficient Dye-Sensitized Solar Cells

**DOI:** 10.1186/s11671-018-2692-1

**Published:** 2018-09-10

**Authors:** Xiuting Luo, Yaojia Zhang, Soo Hyung Kim

**Affiliations:** 1 Department of Nanofusion Technology, Pusan National University, 30 Jangjeon-dong, Geumjung-gu, Busan, 609-735 Republic of Korea; 2 Department of Nano Energy Engineering, Pusan National University, 30 Jangjeon-dong, Geumjung-gu, Busan, 609-735 Republic of Korea; 3 Research Center for Energy Convergence Technology, Pusan National University, 30 Jangjeon-dong, Geumjung-gu, Busan, 609-735 Republic of Korea

**Keywords:** Carbon nanoparticles, Multiwalled carbon nanotubes, Graphene, Dye-sensitized solar cell

## Abstract

Dye-sensitized solar cells (DSSCs) composed of nanostructured carbon composite materials-stacked counter electrodes (CEs) were fabricated in the present study. As the potential replacement of expensive platinum (Pt) thin film, various carbon composite materials, including zero-dimensional carbon nanoparticles (CNPs), one-dimensional multiwalled carbon nanotubes (MWCNTs), and two-dimensional graphene flakes (GFs) as a suitable charge transfer medium were deposited on the surface of CEs using a screen printing process. As the results, CNPs were found to result in deteriorating the charge transfer from CE to liquid electrolyte due to the formation of highly aggregated structures with very low specific surface area. However, MWCNTs and MWCNTs-added carbon composites (e.g., CNP/MWCNT, MWCNT/GF, CNP/MWCNT/GF) were found to enhance the charge transfer from CE to liquid electrolyte due to the formation of highly networked structures with high specific surface area. The resulting PCE of DSSCs composed of pure MWCNTs- and MWCNTs-added carbon composites-based CEs were very similar with that of DSSCs composed of Pt-based CEs. This suggests that the nanostructured carbon materials especially composed of MWCNTs and their composites are one of the promising candidates to replace the expensive Pt in the CEs of DSSCs.

## Background

Dye-sensitized solar cells (DSSCs) have received much attention as an alternative to silicon-based solar cells. They are entitled as one of the most prominent third generation solar cells, because they have the advantages of relatively low manufacturing cost, easy fabrication, and excellent photovoltaic properties [1, 2]. The key components of DSSCs are TiO_2_ thin film-coated fluorine-doped tin oxide (FTO) photoelectrode, dye, liquid electrolyte (${I}^{-}/{I}_3^{-}$ redox couple), and counter electrode (CE) [3, 4].

As an operating principle of DSSC, dye molecules are generally adsorbed on the surface of semiconducting TiO_2_ nanoparticles (NPs) as a photoelectrode. When a DSSC is exposed to sunlight, electrons generated from the excited dye molecules are continuously injected into the conduction band of TiO_2_ NPs, and then they reach the conducting oxide electrode (e.g., FTO glass). The photogenerated electrons are transferred through the external circuit, and then they are introduced into a liquid electrolyte through Pt-coated CEs. The electrolyte finally transports the electrons to complete a current cycle in DSSCs.

As a precious metal, Pt has the advantages of excellent catalytic activity, effective reduction of iodide/triode, and good electrical conductivity so that it is generally employed as CEs of DSSCs [5–11]. However, Pt is relatively expensive, which hinders the massive production of DSSCs in solar cell industry and results in poor stability of DSSCs due to corrosive electrolytes. Thus, many researches have devoted to find out suitable candidates for replacing Pt catalyst in DSSCs with low-cost materials, such as carbon black (CB), carbon nanotube (CNT), alloy metal, metal sulphide, and conducting polymer [5–16]. Among those various alternatives, carbon nanostructured materials such as carbon nanoparticles (CNPs, C_60_), multiwalled carbon nanotubes (MWCNTs), and graphene flakes (GFs) are reported to have potential alternative to Pt in CEs of DSSCs because they have relative high conductivity, large specific surface area, high photochemical stability, and good mechanical strength [17–21].

To fabricate carbon nanostructured materials-coated CEs, various methods, including chemical vapor deposition [22, 23], drop coating [24, 25], spin coating [26], and spray coating process [27] are developed. However, they generally require quite complex fabrication procedures, and simultaneously it is inherently hard to obtain tight bonding and uniform thickness of carbon nanostructured materials employed. Screen-printing is a simple, easy, and versatile process that it makes pressure using a squeegee or other mechanical device to uniformly deposit pastes on the surface of substrate. It can create various printed products with durable property, which can resist to an external contact [28, 29]. Therefore, it has been often employed to make uniform thin films on the surface of substrate, and simultaneously the thickness of thin films can be easily controlled by varying the number of screen-printing process.

In this study, we employ a screen-printing process to fabricate thin films composed of various carbon nanostructured materials, including CNPs, MWCNTs, GFs, and their mixtures on the surface of FTO glass substrates with different thicknesses as CEs of DSSCs. And then, the photovoltaic performance of resulting DSSCs is systematically examined in terms of open-circuit voltage (*V*_oc_), short-circuit current density (*J*_sc_), fill factor (FF), and power conversion efficiency (PCE), which are also finally compared with the photovoltaic performance of Pt-based DSSCs.

## Methods/Experimental

### Fabrication of TiO_2_-Based Photoelectrodes of DSSCs

TiO_2_ NP-based photoelectrode was prepared using a screen-printing process on the surface of FTO glass (SnO_2_: F, 7 Ω/sq., Pilkington, Boston, USA). Commercially available TiO_2_ NPs (P25, Degussa, Germany) were used without further treatment. To fabricate TiO_2_ paste, 6 g of TiO_2_ NPs, 20 g of terpineol, 1 ml of acetic acid (CH_3_COOH), and 15 g of ethanol were mixed in a vial to make a solution-I. And then 3 g of ethyl cellulose and 27 g of ethanol were mixed in another vial to make solution-II. Subsequently, the two solutions were then homogeneously mixed in a vial using a planetary mixer for 3 min, and then it was heated in an oven to remove ethanol. With the assistance of screen-printing process, TiO_2_ thin film was formed on a FTO glass with a photoactive area of 0.6 cm × 0.6 cm with the thickness of ~ 23 μm. The FTO glass was cleaned using acetone, ethanol, and deionized water, and then pretreated with the mixture of 0.247 ml of TiOCl_2_ solution and 20 ml of deionized water to enhance the adhesion between TiO_2_ NPs and FTO glass. The TiO_2_ thin film-coated FTO glass was then sintered at ~ 500 °C for 30 min to remove the residual components. The sintered TiO_2_-coated FTO glass was then immersed into a dye solution containing 0.3 mM of N719 (Solaronix, SA, Switzerland) for 24 h [20].

### Fabrication of Nanostructured Carbon Materials-Based CEs

**Fig. 1:**
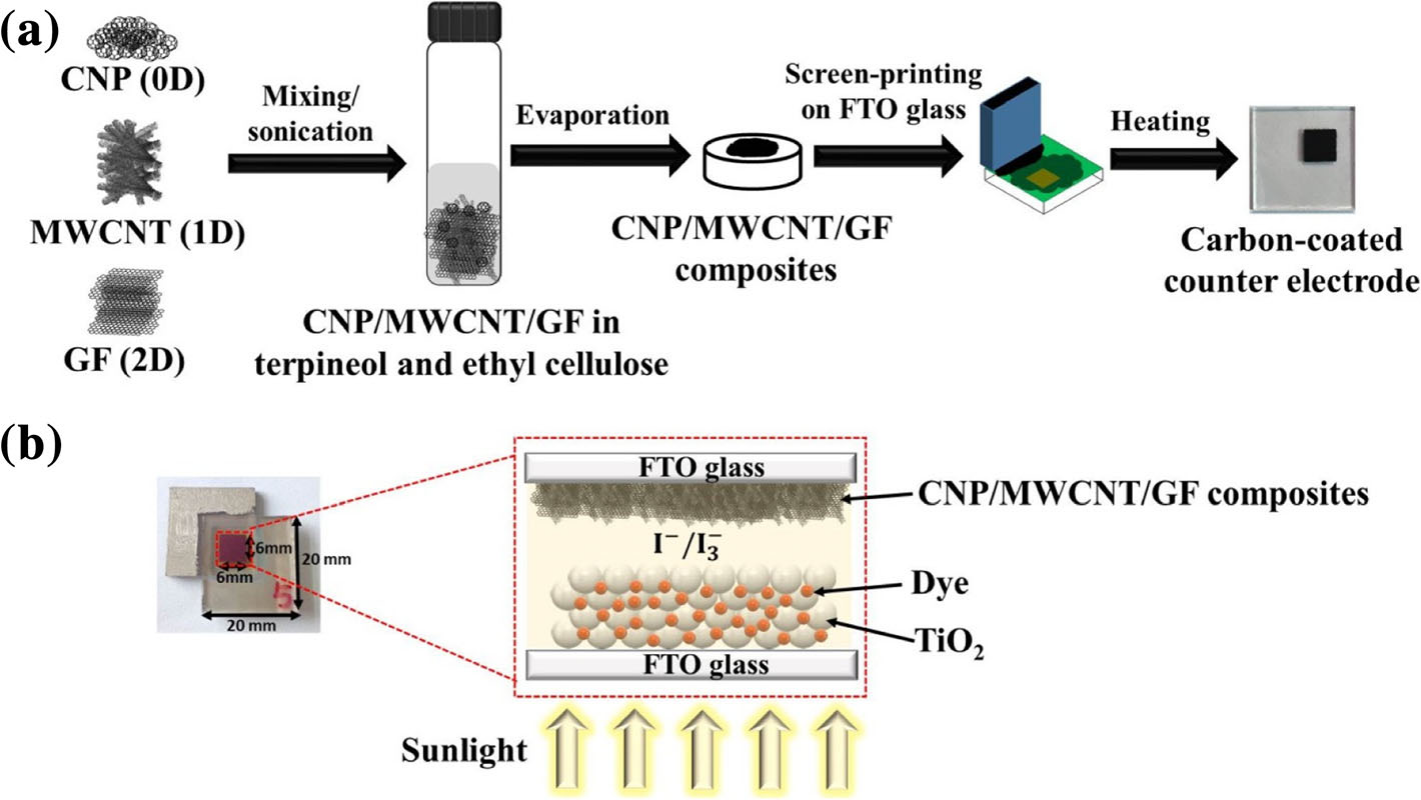
**a** Schematic of fabricating carbon nanoparticle (CNP)/multiwalled carbon nanotube (MWCNT)/graphene flake (GF) composites for counter electrodes (CEs) of dye-sensitized solar cells (DSSCs) and **b** photograph and components of DSSC assembled in the present study

To fabricate a homogeneous CNPs (C_60_, CNT Co., Ltd., Korea), MWCNTs (CNT Co., Ltd., Korea), GFs (CNT Co., Ltd., Korea) paste, 0.2 g of CNPs, 0.2 g of MWCNTs, and 0.2 g of GFs were dispersed in the mixture solution of 1 g of terpineol and 0.1 g of ethyl cellulose, which improved the adhesion between nanostructured carbon materials and substrate. And then they were dispersed into an ethanol solution followed by sonication for 2 h with a probe sonicator (Daihan Scientific Co., Ltd.) to obtain homogenous suspension, which was then evaporated on a hot plate to fabricate a paste with relatively high viscosity. For fabricating various carbon material mixtures, including CNP/MWCNT, CNP/GF/, MWCNT/GF, CNP/MWCNT/GF as shown in Fig. 1a, CNP, MWCNT, and GF powders were dispersed in the solution of terpineol and ethyl cellulose, and then they were treated with sonication and evaporation processes. The seven different pastes composed of CNP, MWCNT, and GF were then screen printed on the surface of FTO glass, which was drilled with two holes with the area of 0.6 cm × 0.6 cm. Then, a heat treatment at 400 ^°^C for 15 min was made to remove any organic contaminants formed on nanostructured carbon materials. The thicknesses of carbon materials employed in the present study were changed by the number of screen-printing process. As a reference CE, a FTO glass was coated with Pt using ion sputter (E1010, Hitachi, Chiyoda-ku, Japan) operated at 1.2 kV and 7 mA.

### Manufacturing and Characterization of DSSCs

The photoelectrodes and CEs fabricated were sealed as a sandwich type configuration with a hot-melt polymer film (60 μm thick, Wooyang, Korea), and then they were heated at 120 ^°^C for 4 min. Subsequently, iodide-based liquid electrolyte (AN-50, Solaronix, SA, Switzerland) was injected into the interspace between two electrodes through the two holes drilled on the CEs, and the holes were then sealed with a cover glass using hot-melt polymer film. Finally, a DSSC unit was completely assembled as shown in Fig. 1b.

The photovoltaic performance of DSSCs fabricated in the present study was measured under air mass 1.5 and 1 sun (=100 mW cm^− 2^) illumination using a solar simulator (PEC-L11, Peccell Technologies, Inc., Kanagawa, Japan). The intensity of light illumination was precisely calibrated using a standard Si photodiode detector with a KG-5 filter. The current density-voltage (J-V) curves and electrochemical impedance spectra (EIS) were recorded automatically with a Keithley SMU 2400 source meter (Cleveland, OH, USA) under illumination of 100 mW cm^− 2^.

The physical structure and thickness of nanostructured carbon materials was measured using a scanning electron microscopy (SEM, S-4200, Hitachi) operated at ~ 15 kV. The specific surface area and porosity was measured using a Brunauer-Emmett-Teller (BET) (ASAP 2020, USA) instrument, and their pore-size distributions were determined by using the Barrett-Joyner-Halenda (BJH) formula from desorption branch. The structural property of nanostructured carbon materials was examined using a Raman spectroscopy (Ramboss 500i, DongWoo Optron), in which a 532 nm laser was used for excitation.

Cyclic voltammetry measurement was performed using an electrochemical workstation of Keithley SMU 2400 source meter (Cleveland, OH, USA) and conventional three-electrode system, which was consisted of carbon composites- or Pt-coated working electrode, a Pt sheet counter electrode and a calomel reference electrode (ALS Co., Ltd., Japan). These electrodes were immersed in 10 mM LiI, 1 mM I_2_ acetonitrile, and 0.1 M LiClO_4_ mixed solution.

## Results and Discussions

**Fig. 2:**
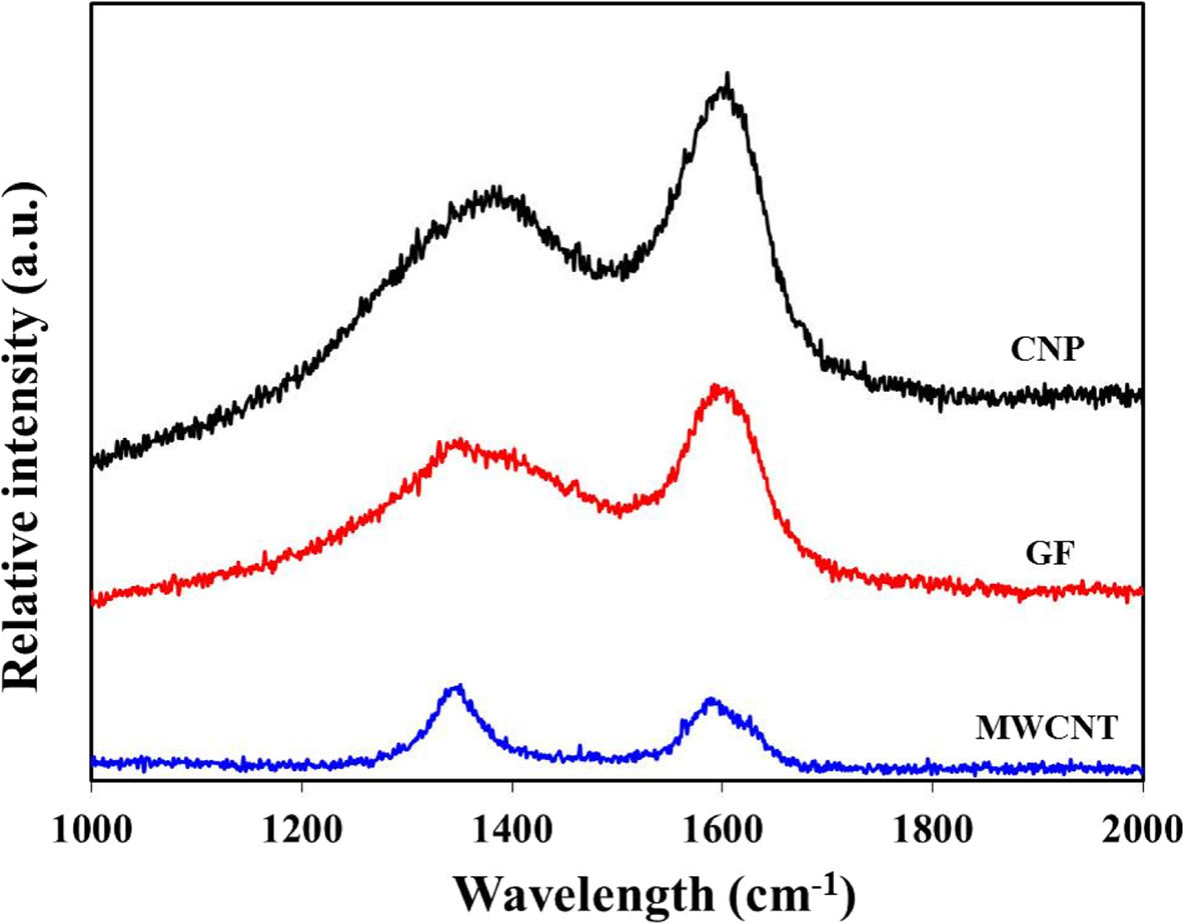
Raman spectra of CNPs, MWCNTs, and GFs

Raman spectroscopy measurement is one of nondestructive analyses for the characterization of crystalline status and defects of carbon materials. Figure 2 shows various Raman spectra for the cases of CNPs, MWCNTs, and GFs. The D peak is related to the first order of zone-boundary phonons and it is known as the disorder peak originated from defects in the carbon material layer. The G peak is the primary mode of carbon materials, and it is known as the planar configuration of sp^2^ bond [13]. The D and G peaks were commonly appeared at 1355 cm^− 1^ and 1579 cm^− 1^ for the CNPs, GFs, and MWCNTs employed in the present study. The relative intensity of D and G peaks (*I*_D_/*I*_G_) indicates the defects of carbon materials [30]. Defects in the nanostructured carbon materials are beneficial for performing an effective catalytic activity because the reduction process of iodide electrolyte in DSSCs occurs at defects in carbon materials [31]. The calculated relative intensity of CNPs, GFs, and MWCNTs were ~ 0.95, ~ 0.97, and ~ 1.01, respectively. The largest relative intensity of D and G peaks was exhibited when MWCNTs are present. It was presumably because MWNCTs have abundant defects in their edge planes. However, it was smaller when CNPs and GFs were present. This was presumably caused by the presence of amorphous structures of CNPs and relatively large 2 D planar structures of GFs, respectively.

**Fig. 3:**
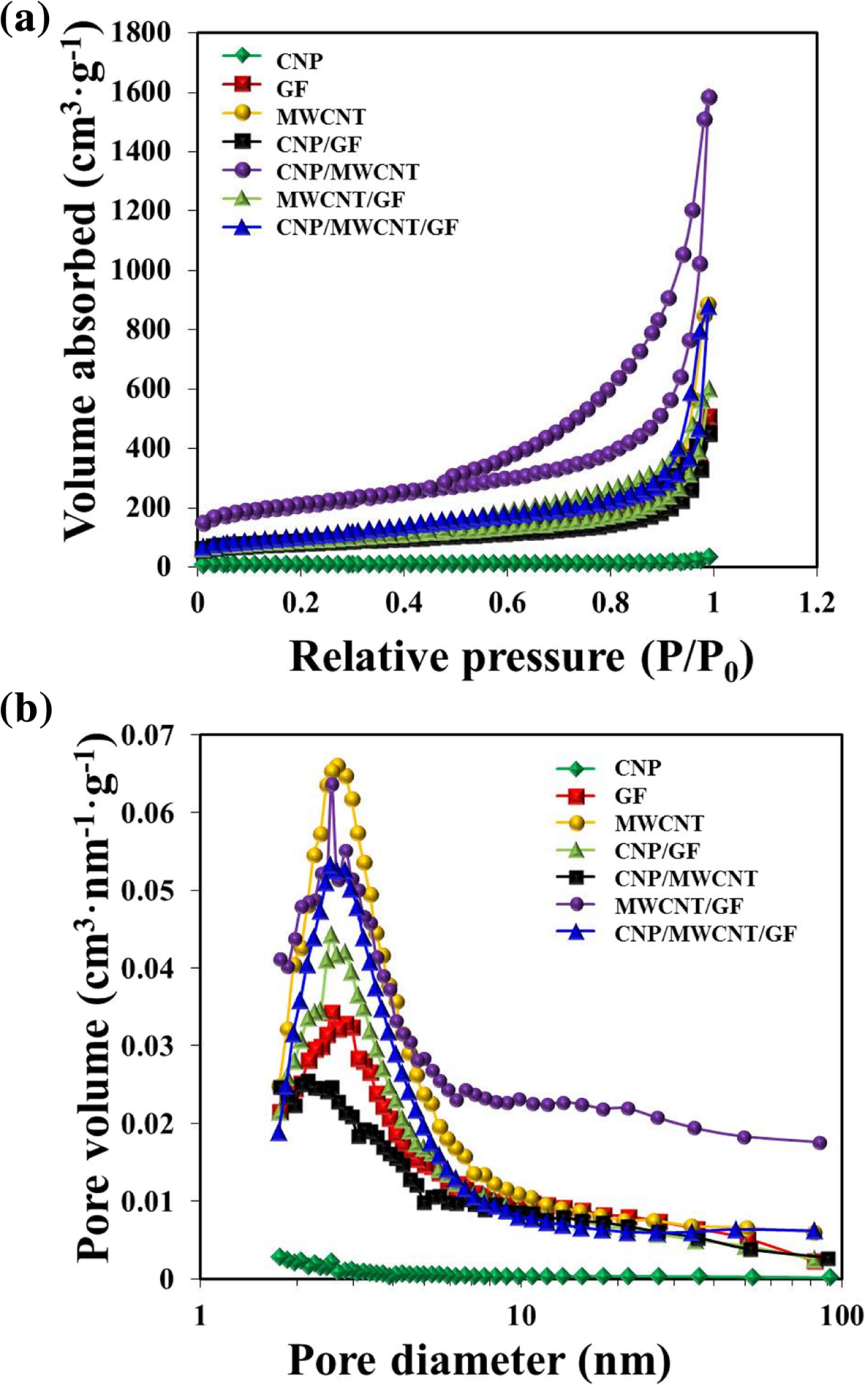
**a** Nitrogen adsorption and desorption curves. **b** Pore volume distributions of CNP, MWCNT, GF, MWCNT/GF, CNP/GF, CNP/MWCNT, and CNP/MWCNT/GF powders

The pore volume distributions of nanostructured carbon materials measured are shown in Fig. 3. The GNPs, MWCNTs, and GFs had the BET surface area of 24.7 m^2^ g^− 1^, 311.8 m^2^ g^− 1^, and 269.5 m^2^ g^− 1^, respectively. The amount of nitrogen adsorbed and the average pore size were increased in the order of CNP/MWCNT > MWCNT > CNP/MWCNT/GF > MWCNT/GF > GF > CNP/GF > CNP, suggesting that the presence of MWCNTs are very effective to increase the specific surface area of nanostructured carbon materials in the CEs of DSSCs so that the electron transfer between CE and liquid electrolyte can be significantly enhanced.

**Fig. 4:**
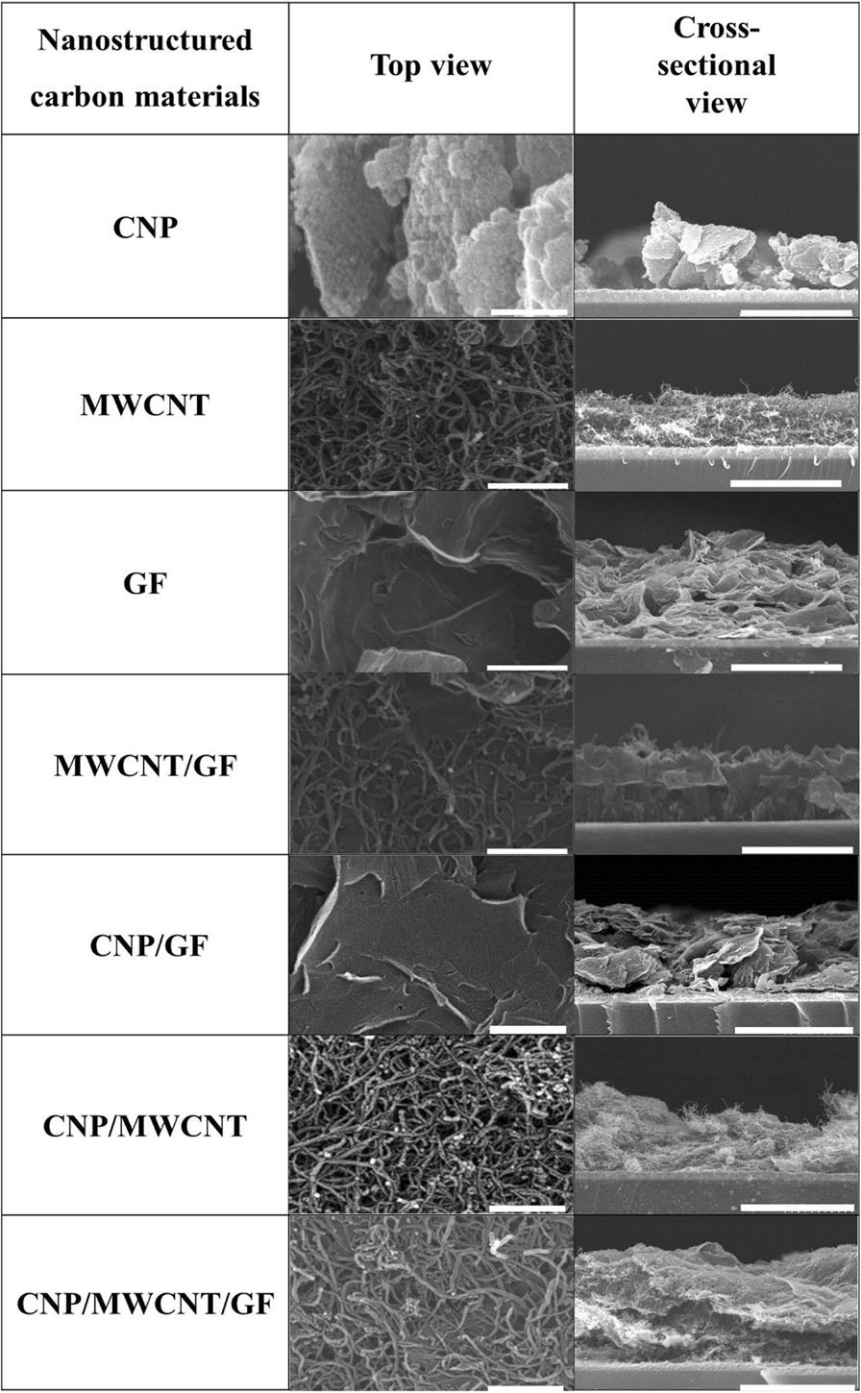
Top- and cross-sectional views of various carbon materials, including CNP, MWCNT, GF, MWCNT/GF, CNP/GF, CNP/MWCNT, CNP/MWCNT/GF stacked on the surface of FTO glass using screen-printing process (the scale bars in the top view images are 0.5 μm, and the scale bars in the cross-sectional view images are 5 μm)

The top view SEM images in Fig. 4 show the morphologies of various nanostructured carbon materials, including CNPs, MWCNTs, GFs, and their composites, which were coated on the surface of FTO lasses. CNPs were seemed to significantly aggregate each other and result in forming clusters separated from the FTO glass, while MWCNTs made randomly networked porous structures, in which ${I}_3^{-}$ ions in liquid electrolyte can easily diffuse to the active sites. The GFs were mostly found to make two-dimensional planar layers. For the case of MWCNT/GF mixture, MWCNT networks were formed on the surface of GFs. After adding CNPs into MWNCTs and GFs, the surfaces of MWCNTs and GFs were partly coated with CNPs. The cross-sectional view SEM images in Fig. 4 clearly show that the CNP-based thin film was not homogeneously bonded to the surface of FTO glass so that interfacial contact between CNPs and FTO glass was very poor. Unlike CNPs, all other nanostructured carbon materials (i.e., CNP/MWCNT, MWCNT/GF, CNP/MWCNT/GF) seemed to have strong attachment to the surface of FTO glass. The thicknesses of nanostructured carbon materials-based thin films were very similar with ~ 5 μm, which can be simply increased with increasing the number of screen-printing process.

**Fig. 5:**
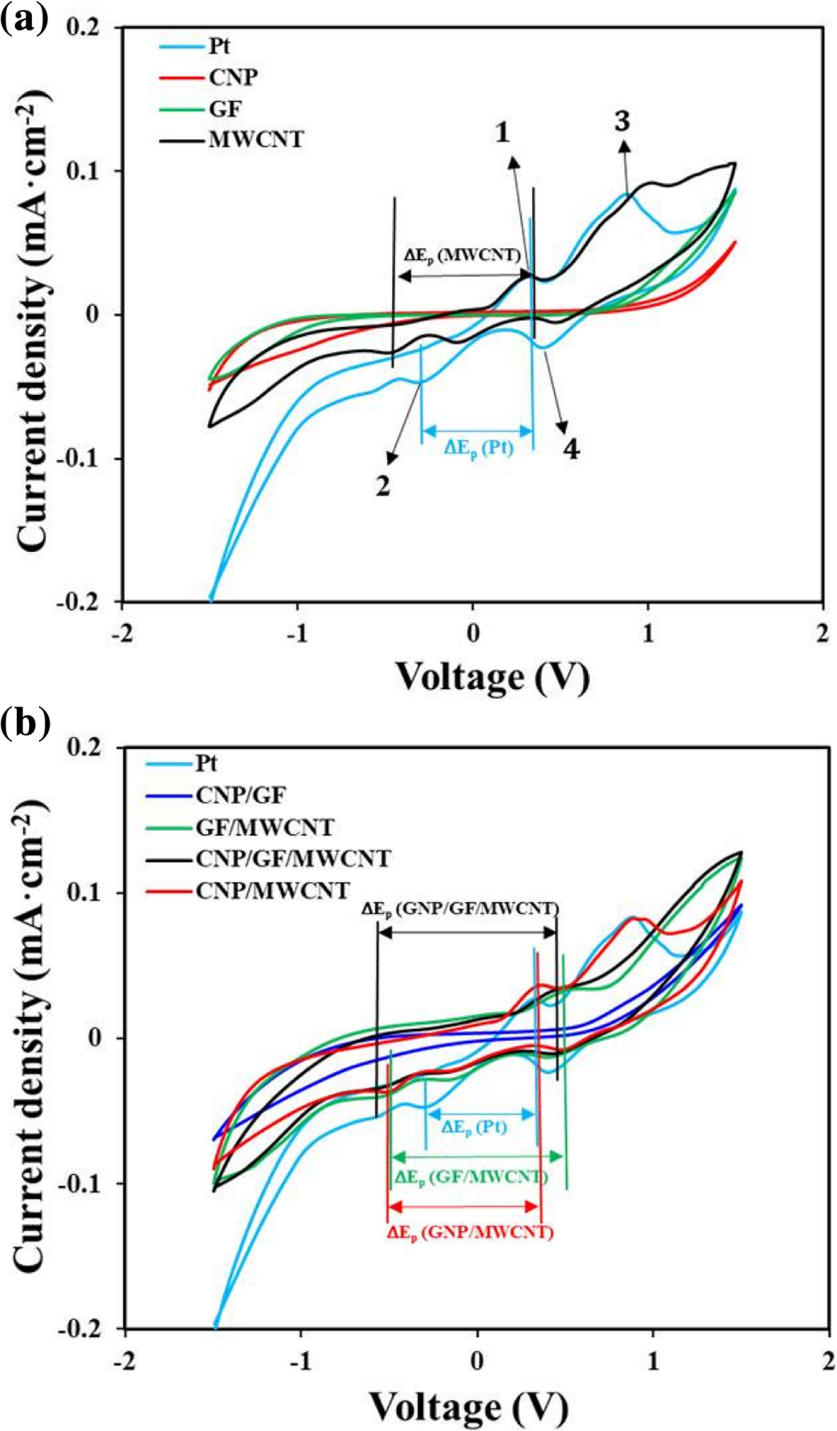
**a** Cyclic voltammetry of Pt-, CNP-, MWCNT-, and GF-coated CEs. **b** Cyclic voltammetry of Pt- and carbon composites-coated CEs measured by scan rate of 50 mV s^− 1^ in 10 mM LiI, 1 mM I_2_ acetonitrile, and 0.1 M LiClO_4_ mixed electrolyte solution

Figure 5 presents the comparison of the cyclic voltammetry curves for I_3_^−^/I^−^ system contacted with the Pt- and carbon material-coated electrodes. Two pairs of oxidation and reduction peaks were clearly observed for Pt and MWCNT cases as shown in Fig. 5a. However, the pure GF and CNP had no clear oxidation and reduction peaks, suggesting that they could not play a key role as potential catalytic materials for CEs of DSSCs. For the cases of Pt and MWCNT-based CEs, the upper and lower peaks in the left-hand side marked as 1 and 2 respectively present the redox reactions expressed in Eqs. (1) and (2), which directly affects the photovoltaic performance of DSSCs. The other two peaks in right-hand side marked as 3 and 4 present the redox reactions expressed in Eqs. (3) and (4), which have a little effect on the photovoltaic performance of DSSCs [12, 32–35].

1 $$ 3{\mathrm{I}}^{-}-2{\mathrm{e}}^{-}=\kern0.5em {\mathrm{I}}_3^{-} $$

2 $$ {\mathrm{I}}_3^{-}\kern0.5em +\kern0.5em 2{\mathrm{e}}^{-}=\kern0.5em 3{\mathrm{I}}^{-} $$

3 $$ 2{\mathrm{I}}_3^{-}-2{\mathrm{e}}^{-}=3{\mathrm{I}}_2 $$

4 $$ 3{\mathrm{I}}_2+2{\mathrm{e}}^{-}=2{\mathrm{I}}_3^{-} $$

In DSSCs, the photogenerated electrons are transferred from I^−^ ions in the electrolyte to photo-oxidized dye, and the ${\mathrm{I}}_3^{-}$ ions are reduced on the surface of CEs. In the CV curves, the peak-to-peak separation was observed to be varied inversely with charge transfer rate [34, 35]. Figure 5a shows that the redox peaks for Pt-coated CEs were appeared at − 0.29 V and 0.33 V, respectively, and the resulting *∆*E_p_(Pt) was ~ 0.62 V. In contrast, the redox peaks for MWCNT-coated CEs were appeared at − 0.44 V and 0.33 V, respectively, and the resulting *∆*E_p_(MWCNT) was ~ 0.77 V. As shown in Fig. 5b, for the cases of MWCNT-added carbon composites-based CEs, the resulting ΔE_p_(CNP/MWCNT), ΔE_p_ (CNP/GF/MWCNT), and ΔE_p_(GF/MWCNT) were ~ 0.83 V, ~ 0.98 V, and ~ 1.025 V, respectively. This suggests that pure MWCNTs- and MWCNT-added carbon composites-based CEs had relatively high catalytic activity and rapid reaction rate in triiodide reduction. The presence of MWCNTs were very effective to increase the specific surface area of nanostructured carbon materials in the CEs of DSSCs so that the electron transfer between carbon composites-coated CE and liquid electrolyte was significantly enhanced.

**Fig. 6:**
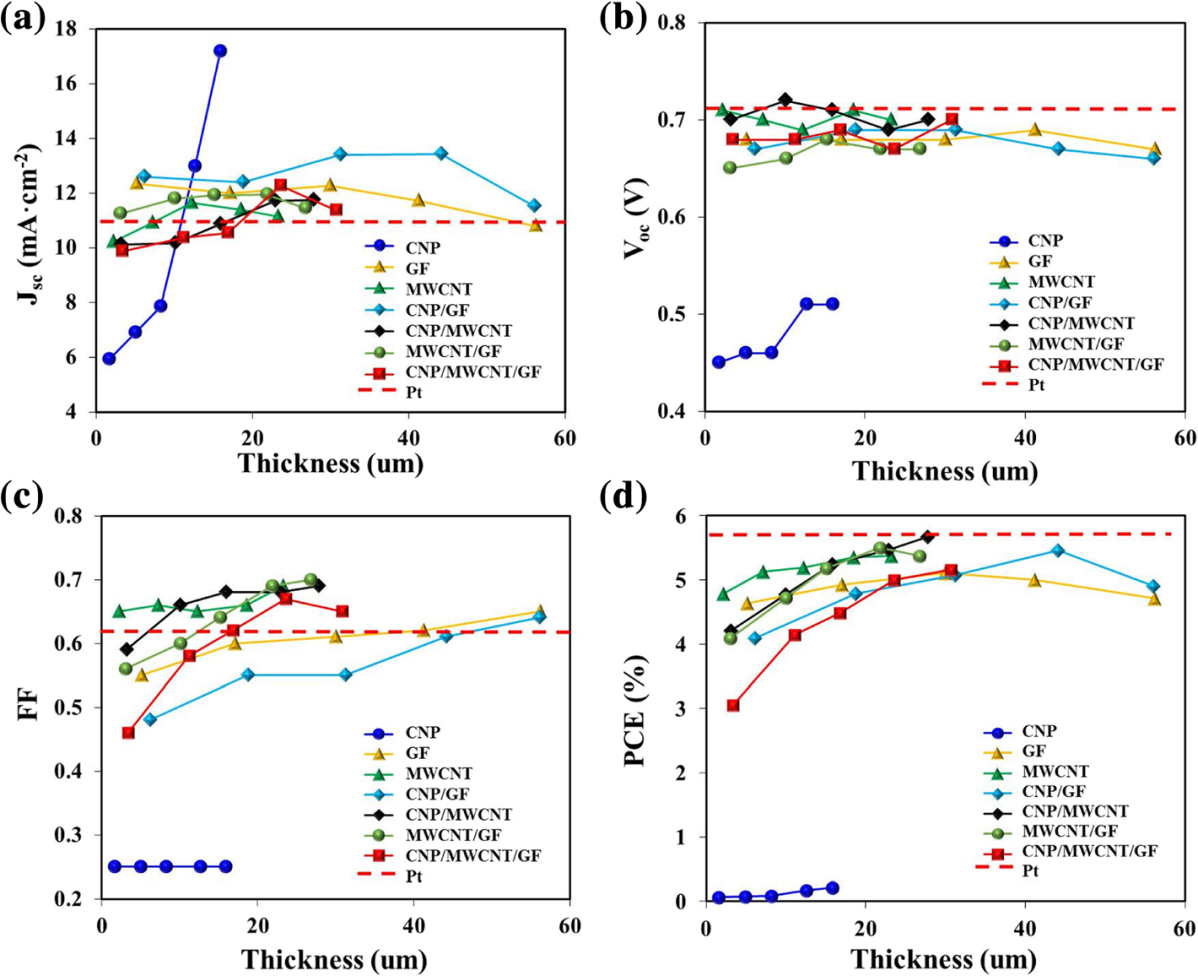
The comparison of photovoltaic performances of DSSCs composed of various carbon materials- and Pt-based CEs in terms of **a** *J*_sc_, **b** *V*_oc_, **c** FF, and **d** PCE

Figure 6 exhibits the resulting photovoltaic performance of DSSCs in terms of short-circuit current density (*J*_sc_), open-circuit voltage (*V*_oc_), fill factor (FF), and power conversion efficiency (PCE) as a function of the thickness of nanostructured carbon materials in CEs of DSSCs. For the case of CNPs, *J*_sc_ was significantly increased with increasing the thickness of CNP thin film, but both FF and *V*_oc_ were not appreciably changed in relatively low values, which finally resulted in very poor PCE values. This must be occurred by the formation of severe clusters between CNPs so that the electrons were effectively transported from CEs to liquid electrolyte. For the cases of GF and CNP/GF, FFs were also relatively poor. This was presumably because the 2D planar structures of GFs were crumpled and twisted to some extent so that they were not intimately contacted with each other in the stacking arrangement. Therefore, the resulting PCEs of DSSCs made by GF- and CNP/GF-based CEs were relatively low. However, the presence of MWCNTs in the nanostructured carbon materials (i.e., MWCNT, MWCNT/GF, CNP/MWCNT, CNP/MWCNT/GF) was observed to stably increase the *J*_sc_ and FF so that the resulting PCEs of DSSCs were maintained in relatively high values. This was presumably because the intimate networks and high specific surface area formed by the presence of MWCNTs enhanced the electron transport at the interface of CE and liquid electrolyte.

**Fig. 7:**
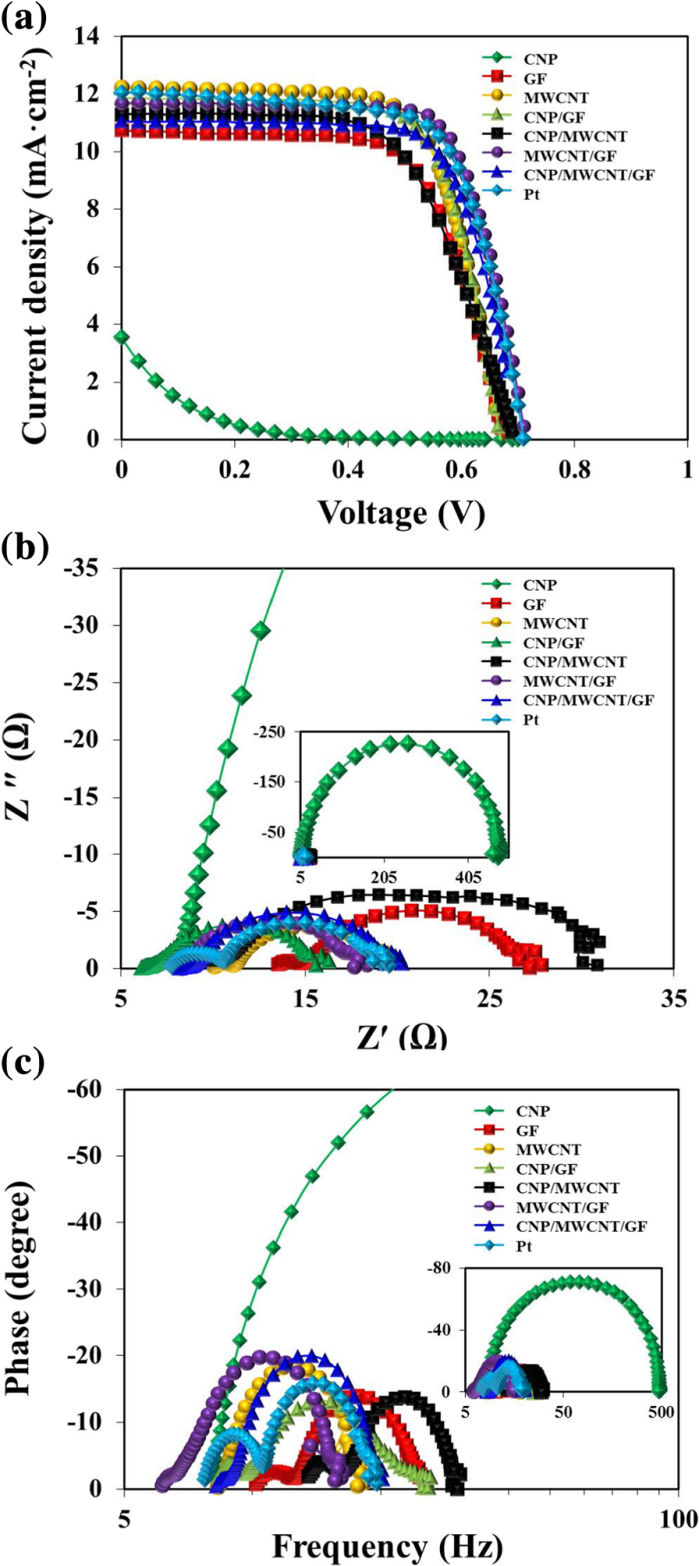
Comparison of **a** current density-voltage curves, **b** Nyquist plots, and **c** Bode plots for the DSSCs composed of various carbon materials- and Pt-based CEs

The current density-voltage (J-V) and electrochemical impedance spectroscopy (EIS) measurements were performed for the CEs stacked with different carbon materials with the similar thickness of ~ 20 μm as shown in Fig. 7a and Table 1. Those of conventional Pt-based CEs were also performed for comparison. The DSSCs stacked with CNPs in CEs had extremely high *J*_sc_ of ~ 17.18 mA cm^− 2^, but quite low *V*_oc_ of ~ 0.5 V and FF of ~ 0.25, thus it caused the lowest PCE of ~ 0.22%, suggesting that CNPs are not suitable for DSSCs due to strong aggregation-induced low interfacial contact area with FTO glass in the CEs. The DSSCs stacked with GF and CNP/GF in CEs also showed lower FF and PCE due to their relatively low specific surface area confirmed by previous BET measurements as shown in Fig. 3. However, the DSSCs stacked with MWCNT and MWCNT-added carbon composite materials had higher PCEs of > 5%. The DSSCs stacked with CNP/MWCNT composites had the best PCE of ~ 5.67%, which was very close to the PCE of ~ 5.7% generated by the Pt-based DSSCs. This suggests that the higher specific surface area created by employing MWCNT-based nanostructured carbon composite materials promoted the reduction process more effectively at the interface of CE and liquid electrolyte. Figure 7b shows Nyquist plots for the DSSCs composed of various carbon materials-based CEs. The transport resistance (*R*_ce_) is related to the first semicircle and interfacial capacitance (CPE_pt_), which is the charge transfer at CEs. The recombination resistance (*R*_rec_) and interfacial capacitance (CPE_TiO2/dye/electrolyte_) are related to the second semicircle, which represent the charge transfer at the interfaces of TiO_2_/dye/electrolyte [36–38]. Table 1 shows that DSSCs composed of all the carbon materials except CNP and CNP/CF composite cases employed in the present study exhibited lower *R*_ce_ than that of Pt-based DSSCs, indicating that MWCNTs and their composites had high electrocatalytic reactivity and electrical conductivity, and thus there were fewer losses of electrons at the interface of CE and liquid electrolyte. And *R*_rec_ was decreased with increasing the specific surface area of carbon materials, which eventually resulted in reducing the electron recombination in the interface of dye and electrolyte. However, the value of *R*_rec_ for Pt-based DSSCs was much lower than that of carbon material-based DSSCs, suggesting that the Pt was more beneficial to the charge transfer at the interfaces of TiO_2_/dye/electrolyte, and carbon materials were not able to rapidly reduce the ${\mathrm{I}}_3^{-}$ compare with Pt [39]. Figure 7c shows Bode plots for the DSSCs composed of various carbon materials. The electron lifetime (*τ*_*e*_) can be calculated by τ_e_ = (2π*f*_max_)^− 1^ (where, *f*_max_ is the maximum peak frequency) [40]. When MWCNTs were present in the carbon composite materials, the electron lifetime of carbon material-based DSSCs was longer than that of Pt-based DSSCs. This suggests that the electrons were diffused further due to rapid charge transfer from CEs to liquid electrolyte through the MWCNTs and MWCNTs-added carbon composites, which had inherently higher specific surface area.

**Table 1:** Photovoltaic performance of DSSCs based on different catalytic materials stacked on the surface of CEs of DSSCs

Catalytic materials in CEs*	SSA* (m^2^ g^− 1^)	Thickness (μm)	*J*_sc_* (mA cm^− 2^)	*V*_oc_* (V)	FF*	PCE* (%)	*R*_rec_*(Ω)	*R*_ce_*(Ω)	τ_e_* (ms)
CNP*	24.70	20.85	17.18	0.50	0.25	0.22	354.10	117.93	2.20
CNP/GF*	252.12	21.12	11.54	0.68	0.61	4.72	15.86	3.30	6.34
GF	269.51	21.23	11.72	0.69	0.62	4.93	13.10	2.50	7.96
MWCNT*/GF	303.76	23.21	11.14	0.70	0.69	5.37	10.90	2.39	9.05
CNP/MWCNT/GF	305.60	23.56	11.90	0.67	0.67	5.49	9.13	1.00	11.54
MWCNT	311.83	21.80	12.28	0.67	0.69	5.50	9.06	0.34	12.63
CNP/MWCNT	356.73	22.82	11.75	0.70	0.69	5.67	8.00	0.14	15.31
Pt	–	–	11.93	0.71	0.67	5.70	7.80	2.88	11.20

*Note: *CE* counter electrode, *CNP* carbon nanoparticle, *GF* grapheme flake, *MWCNT* multiwalled carbon nanotube, *SSA* specific surface area, *J*_sc_: short circuit current, *V*_oc_ open circuit voltage, *FF* fill factor, *PCE* power conversion efficiency, *R*_rec_ recombination resistance, *R*_ce_ resistance of counter electrode, *τ*_*e*_ electron lifetime

## Conclusions

In this work, we systematically examined the effect of various nanostructured carbon materials as a Pt replacement in CEs on the photovoltaic performance of DSSCs. Specifically CNPs, MWCNTs, GFs, and their composites were stacked on the surface of CEs, and the resulting photovoltaic performance of DSSCs were measured in terms of *J*_sc_, *V*_oc_, FF, and PCE. As the results, CNPs were not suitable for using as Pt replacement in the CEs of DSSCs due to the formation of highly aggregated structures, which resulted in detaching the formed CNP-based thin film from the surface of FTO glass. Unlike CNPs, the presence of MWCNTs in the various carbon composites was found to effectively promote the charge transfer from CEs to liquid electrolyte due to the formation of highly networked MWCNT structures with inherently high specific surface area on the surface of FTO glass. Therefore, the nanostructured carbon materials especially composed of MWCNTs and MWCNTs-added carbon composites (e.g., CNP/MWCNT, MWCNT/GF, CNP/MWCNT/GF) are one of promising candidates to replace the expensive Pt in the CEs of DSSCs.

## Abbreviations

BET: Brunauer-Emmett-Teller
CEs: Counter electrodes
CNPs: Carbon nanoparticles
DSSCs: Dye-sensitized solar cells
EIS: Electrochemical impedance spectroscopy
FF: Fill factor
FTO: Fluorine-doped tin oxide
GFs: Graphene flakes
MWCNTs: Multiwalled carbon nanotubes
PCE: Power conversion efficiency
SEM: Scanning electron microscopy
